# p53-mediated AKT and mTOR inhibition requires RFX7 and DDIT4 and depends on nutrient abundance

**DOI:** 10.1038/s41388-021-02147-z

**Published:** 2021-12-14

**Authors:** Luis Coronel, David Häckes, Katjana Schwab, Konstantin Riege, Steve Hoffmann, Martin Fischer

**Affiliations:** Computational Biology Group, Leibniz Institute on Aging – Fritz Lipmann Institute (FLI), Beutenbergstraße 11, 07745 Jena, Germany

**Keywords:** Oncogenes, TOR signalling, Nutrient signalling, Stress signalling, Gene regulation

## Abstract

In recent years the tumor suppressor p53 has been increasingly recognized as a potent regulator of the cell metabolism and for its ability to inhibit the critical pro-survival kinases AKT and mTOR. The mechanisms through which p53 controls AKT and mTOR, however, are largely unclear. Here, we demonstrate that p53 activates the metabolic regulator DDIT4 indirectly through the regulatory factor X 7 (RFX7). We provide evidence that DDIT4 is required for p53 to inhibit mTOR complex 2 (mTORC2)-dependent AKT activation. Most strikingly, we also find that the DDIT4 regulator RFX7 is required for p53-mediated inhibition of mTORC1 and AKT. Our results suggest that AMPK activation plays no role and p53-mediated AKT inhibition is not critical for p53-mediated mTORC1 inhibition. Moreover, using recently developed physiological cell culture media we uncover that basal p53 and RFX7 activity can play a critical role in restricting mTORC1 activity under physiological nutrient conditions, and we propose a nutrient-dependent model for p53-RFX7-mediated mTORC1 inhibition. These results establish RFX7 and its downstream target DDIT4 as essential effectors in metabolic control elicited by p53.

## Introduction

The pro-survival kinase mTOR (mammalian target of rapamycin) supports tumor development and is frequently activated in cancer [1]. The mTOR kinase is the catalytic subunit of two distinct complexes, mTOR complex 1 and 2 (mTORC1 and mTORC2), and pro-survival properties are largely attributed to mTORC1. The best-described signaling pathway inducing mTORC1 involves the triggering of PI3K (phosphatidylinositol-4,5-bisphosphate 3-kinase) by growth factors and other external stimuli. Subsequently, the protein kinase AKT (also known as protein kinase B; PKB) is activated through phosphorylation at Thr308 and Ser473 by PDK1 (pyruvate dehydrogenase kinase 1) and mTORC2, respectively [2]. When activated, AKT phosphorylates and deactivates the mTORC1 inhibitors TSC2 and PRAS40, leading to mTORC1 activation [1, 2]. While PI3K-AKT-signaling activates mTORC1 in response to growth stimuli, AMPK (adenosine monophosphate-activated protein kinase) senses energy levels and inhibits mTORC1 when energy supply is low. AMPK comprises α, β, and γ subunits, and the phosphorylation at Thr172 of AMPKα is critical for its activity [3]. When activated, AMPK can inhibit mTORC1 through inducing TSC2 and repressing RAPTOR [1, 3]. Thus, PI3K-AKT-mTORC1 and AMPK-mTORC1 are two major signaling pathways regulating mTORC1 in response to growth factors and energy levels, respectively.

The tumor suppressor p53 is a well-known inhibitor of mTORC1 in mouse and human [4–7]. Moreover, p53 has been shown to inhibit AKT [8, 9] and to activate AMPK [5, 6]. In the case of AKT, p53 inhibits the activating pSer473 mark, which is mediated by mTORC2 [8, 9]. However, other reports indicate that p53 also can activate AKT [10] and does not affect AMPK [11]. p53 transcriptionally up-regulates multiple regulators of the PI3K-AKT-mTORC1 and AMPK-mTORC1 pathways, such as the PI3K opponent PTEN [8], the AKT inhibitors PHLDA3 and ASS1 [9, 12], and the negative mTORC1 regulators AMPKβ [13], SESN1, SESN2 [6], and DEPTOR [14]. Consequently, the inhibition of mTORC1 activity is perceived to be critical for p53’s ability to regulate cell fitness [15] and to suppress tumorigenesis [16–18]. Most recently, it has been hypothesized that p53-mediated activation of the known mTORC1 inhibitors SESN1, SESN2, and DDIT4 may be of particular relevance [17]. DDIT4 (also known as REDD1) is a p53 target [19] that has been shown to inhibit both AKT and mTORC1 [20–22]. While it has not been assessed whether p53-induced DDIT4, PTEN, AMPKβ, ASS1, and DEPTOR inhibit mTORC1 or AKT [8, 12–14, 19], knockdown of PHLDA3 has been shown to increase AKT activity irrespective of p53 [9]. The p53 targets SESN1 and SESN2 were essential for p53-mediated mTORC1 inhibition in MEF but only contributed to it in U2OS cells. Mechanistically, for both proteins it was suggested that the inhibition of mTORC1 is achieved via AMPK and TSC2 [6]. More recent studies, however, showed that SESN1/2 control mTORC1 through GATOR2 instead of AMPK and TSC2 in both mouse and human cells [23, 24]. Likewise, also p53 has been suggested to inhibit mTORC1 independent of AMPK [11]. Previously, we uncovered that the p53 gene regulatory network differs markedly between mouse and human, and in particular with regard to controlling metabolism-associated genes [25, 26]. For example, *PRKAA1* (encoding AMPKα1) is a direct p53 target only in mice. On the other hand, *PTEN*, *SESN1*, *PRKAB1* (AMPKβ1), and *PRKAB2* (AMPKβ2) are direct p53 targets only in humans [25]. Despite these findings, the mechanisms through which p53 inhibits mTORC1 and AKT in human cells remain surprisingly poorly understood. Here, we aimed to unravel the mechanisms through which p53 inhibits mTORC2-dependent AKT activity and mTORC1.

The emerging tumor suppressor RFX7, a putative cancer driver in Burkitt lymphoma [27, 28] that was previously associated with multiple lymphoid neoplasms [29], up-regulates *Ddit4* and inhibits mTORC1 activity in murine lymphoid cells [30]. Most recently, we found that p53 activates RFX7 through the induction of a lower migrating form, and we identified *DDIT4* as a potential target of RFX7 also in human cells [31]. We now demonstrate that DDIT4 is indirectly up-regulated by p53 through the novel tumor suppressor RFX7 in human cell line models. Our study reveals that DDIT4 is required for p53-mediated inhibition of mTORC2-dependent AKT activity. Surprisingly, the DDIT4 regulator RFX7 is required for p53-mediated inhibition of both AKT and mTORC1. The p53-RFX7 signaling axis inhibits mTORC1 in a nutrient-dependent manner.

## Results

### RFX7 mediates DDIT4 activation by p53 and stress

**Fig. 1:**
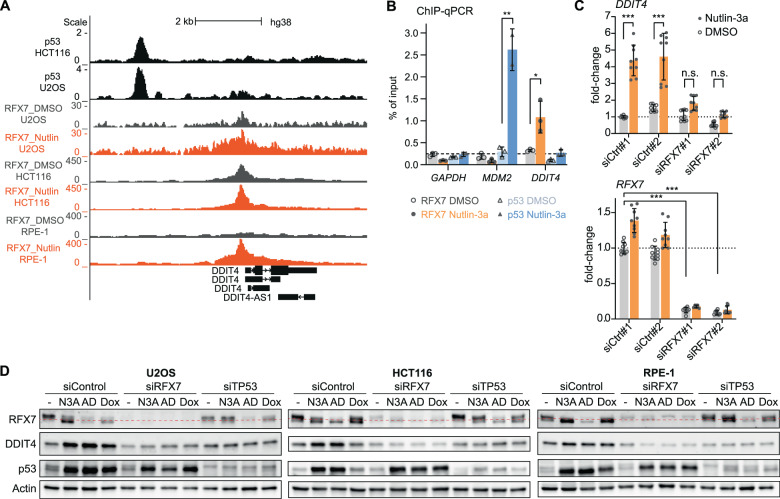
RFX7 mediates DDIT4 up-regulation by p53 and stress. **A** Genome browser snapshot of the *DDIT4* gene locus. Upper black tracks display publicly available p53 binding signals from Nutlin-3a-treated U2OS [40] and HCT116 [39] cells. Gray and orange tracks display RFX7 binding signals from respective dimethyl sulfoxide (DMSO) and Nutlin-3a-treated U2OS, HCT116, and RPE-1 cells [31]. **B** ChIP-qPCR of p53 and RFX7 binding to *GAPDH* (negative control), *MDM2* (p53 positive control), and *DDIT4* from U2OS cells treated with 10 µM Nutlin-3a or DMSO solvent control. Mean and standard deviation is displayed. Statistical significance obtained through a two-sided unpaired *t*-test, *n* = 3 technical replicates. **C** RT-qPCR data of *DDIT4* and *RFX7* in 10 µM Nutlin-3a and DMSO control-treated U2OS cells transfected with two different control siRNAs (siCtrl) and two different siRNAs against *RFX7*. Normalized to siControl#1 DMSO and *ACTR10* control gene. Mean and standard deviation is displayed. Statistical significance obtained through a two-sided unpaired *t*-test, *n* = 9 replicates (three biological with three technical each). **D** Western blot analysis of RFX7, DDIT4, p53, and actin (loading control) levels in U2OS, HCT116, and RPE-1 cells transfected with siControl, siRFX7, or siTP53 and treated with DMSO solvent control, 10 µM Nutlin-3a (N3A), 5 nM Actinomycin D (AD), and 1 µM Doxorubicin (Dox).

*DDIT4* has been established as a p53-responsive gene [19], but there are conflicting data regarding the underlying mechanism. Reporter gene assays indicated that a putative p53 responsive element immediately upstream of *DDIT4*’s transcriptional start site (TSS) is crucial for p53-mediated *DDIT4* induction [19], but p53 ChIP-seq data show that p53 instead binds 3 kb upstream of the TSS (Fig. 1A). However, reporter gene assays did not confirm p53-mediated activation through this locus [19]. We recently identified *DDIT4* as a potential target of the novel p53-RFX7 signaling pathway [31]. Given these conflicting data, we investigated whether *DDIT4* induction by p53 is mediated indirectly via RFX7. Examining recently published RFX7 ChIP-seq data revealed RFX7 binding immediately upstream of *DDIT4*, and showed that RFX7 occupancy increased upon Nutlin-3a treatment (Fig. 1A). We assessed the binding of p53 and RFX7 to the *DDIT4* promoter by ChIP-qPCR. In contrast to the well-established p53-target MDM2, the proximal promotor of *DDIT4* displayed no p53 binding above background control. However, the new p53-target RFX7 binds specifically to the *DDIT4* promoter and the binding is significantly induced upon Nutlin-3a treatment (Fig. 1B). To test whether RFX7 regulates *DDIT4* and its response to p53, we employed RT-qPCR using two siRNAs for RFX7 depletion and control to exclude off-target effects. Both siRNAs mediated efficient knockdown of *RFX7* and significantly impaired Nutlin-3a-mediated *DDIT4* induction (Fig. 1C). To assess p53-dependent regulation of DDIT4 protein levels, we used the topoisomerase II inhibitor Doxorubicin and the rRNA transcription inhibitor Actinomycin D in addition to the MDM2 inhibitor Nutlin-3a, all of which are well-established inducers of p53 signaling [32]. Immunoblots showed that Nutlin-3a, Actinomycin D, and Doxorubicin induced p53 in U2OS, HCT116, and RPE-1 cells. In addition RFX7 was activated, as indicated by its lower migrating band. Most importantly, both p53 and RFX7 were required for the induction of DDIT4 protein levels (Fig. 1D). Together, these results demonstrate that p53 up-regulates DDIT4 indirectly through RFX7.

### The DDIT4 regulator RFX7 is required for mTORC1 inhibition by p53

**Fig. 2:**
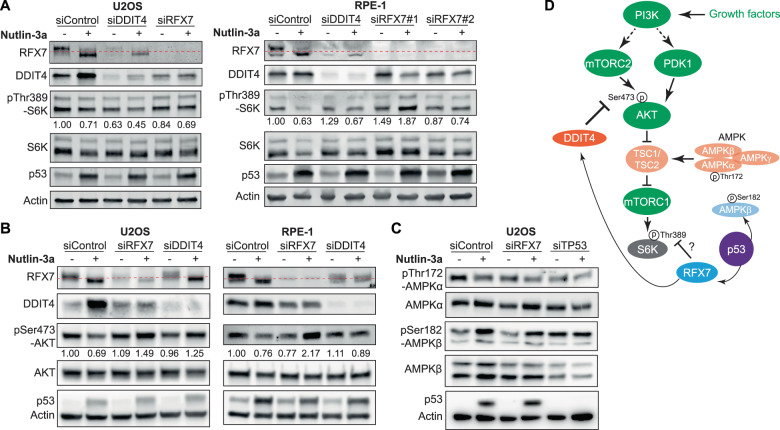
p53 inhibits AKT and mTOR through DDIT4 and RFX7. **A–C** Western blot analysis of U2OS and RPE-1 cells transfected with indicated siRNAs and treated with 10 µM Nutlin-3a or DMSO control. Actin served as loading control. Densitometric quantification relative to siControl DMSO samples and actin levels. **D** Critical nodes in p53-dependend AKT and mTORC1 control. Blue nodes are direct p53 target genes in human. Black edges indicate activation or inhibition. Green and orange nodes are established activators and inhibitors of mTORC1, respectively, irrespective of p53. Saturated blue and orange nodes (RFX7 and DDIT4) were assessed for their p53-dependent mTORC1 control in this study. p53 appears to inhibit mTORC2-AKT signaling through RFX7-DDIT4. RFX7 is required for p53-mediated AKT and mTORC1 inhibition. RFX7 employs yet unknown targets to inhibit mTORC1 (indicated by a question mark).

Given that inhibition of mTORC1 has been observed for DDIT4 [20, 22] and mouse Rfx7 [30], we assessed the effect of DDIT4 and RFX7 on p53-dependent mTORC1 activity in human cells. Nutlin-3a treatment induced p53 and the lower migrating form of RFX7 and inhibited mTORC1 activity, measured by pThr389 of mTORC1’s key target S6K. While DDIT4 is a well-established inhibitor of mTORC1 [20, 22], the complex’s activity was essentially not elevated upon DDIT4 depletion in both Nutlin-3a and DMSO control-treated U2OS and RPE-1 cells. Induction of p53 led to a reduction in mTORC1 activity also when DDIT4 was depleted (Fig. 2A). These results suggest that DDIT4 is not required for p53-mediated mTORC1 inhibition. Intriguingly, depletion of RFX7 substantially reduced Nutlin-3a-induced mTORC1 inhibition (Fig. 2A), indicating that RFX7 is required for p53 to inhibit mTORC1.

### DDIT4 and RFX7 are required for p53-mediated mTORC2-AKT inhibition

Given that the mTORC1 activator AKT was shown to be inhibited by p53 [8, 9], we assessed whether its activity correlates with p53-regulated mTORC1 activity. Notably, in insulin-treated mouse fibroblasts and upon ectopic expression in HEK293 cells, DDIT4 was shown to inhibit AKT by promoting Thr308 de-phosphorylation through PP2A, but to not affect mTORC2-mediated Ser473 phosphorylation [21]. Induction of p53 by Nutlin-3a indeed led to reduced pSer473-AKT levels in both U2OS and RPE-1 cells. Surprisingly, p53-dependent inhibition of AKT, measured by its Ser473 phosphorylation, was lost upon depletion of DDIT4 and RFX7 (Fig. 2B), indicating that both DDIT4 and RFX7 are required for p53 to inhibit mTORC2-AKT signaling. Moreover, these findings suggest that p53-dependent AKT and mTORC1 inhibition are not strictly linked but can occur independent of another.

### AMPK activation is not mandatory for mTORC1 inhibition by p53

Next, we assessed the activity of the negative mTORC1 regulator AMPK. Notably, Feng et al. used Compound C to inhibit AMPK and found AMPK activity to be essential for p53’s effect on mTORC1 in MEF and human V138 cells [5]. Although Compound C is not AMPK-specific but inhibits numerous kinases [33], a knockdown of AMPKα1 in H1299 cells corroborated the hypothesis that AMPK might indeed contribute to p53-mediated mTORC1 inhibition [6]. Upon Nutlin-3a treatment, p53 rather inhibited AMPK in U2OS cells, as indicated by reduced pThr172-AMPKα levels (Fig. 2C), suggesting that AMPK activation is not mandatory for p53-mediated mTORC1 inhibition. RFX7 knockdown did not affect AMPK activity either, indicating that RFX7 does not employ AMPK to inhibit mTORC1. Given that p53 up-regulates *PRKAB1* (AMPKβ1) and *PRKAB2* (AMPKβ2) instead of *PRKAA1* (AMPKα1) in humans [25], we tested whether AMPKβ was affected by p53. Nutlin-3a treatment indeed led to a p53-dependent and RFX7-independent increase of pSer182-AMPKβ levels (Fig. 2C).

Together, these data establish that p53 requires RFX7 to inhibit both AKT and mTORC1, and p53-RFX7-mediated inhibition of mTORC1 is not associated with AMPK activation. While the RFX7 target DDIT4 could be an explanation for p53-RFX7-mediated AKT inhibition, a contribution to p53-RFX7-mediated mTORC1 inhibition was not evident. Thus, other RFX7 targets may regulate mTORC1 activity (Fig. 2D).

### p53 and RFX7 limit mTORC1 activity under physiological glucose and glutamine levels

**Fig. 3:**
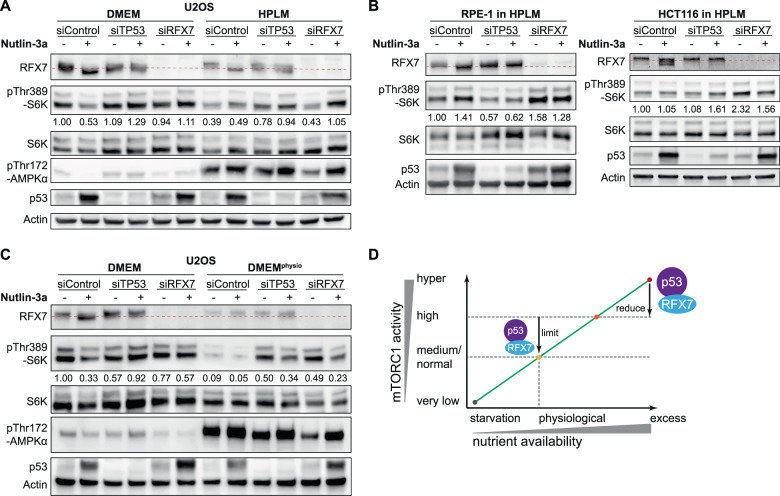
Nutrient-dependent inhibition of mTORC1 by p53 and RFX7. Western blot analysis of U2OS cells transfected with indicated siRNAs, treated with 10 µM Nutlin-3a or DMSO control, and cultured in DMEM and (**A**) HPLM or (**C**) DMEM^physio^. A complementary replicate of (**A**) with additional measurements is available through Supplementary Fig. 1. **B** Western blot analysis of RPE-1 and HCTT116 cells transfected with indicated siRNAs, treated with 10 µM Nutlin-3a or DMSO control, and cultured in HPLM. Densitometric quantification relative to siControl DMSO samples and actin levels. **D** Nutrient-dependent phase model of p53-RFX7-mediated mTORC1 inhibition. The low mTORC1 activity we observed in U2OS cells cultured with physiological nutrient access likely resembles rather normal/medium activity levels, whereas the elevated mTORC1 activity in cells cultured with excess nutrients reflects high or hyper-activity. Both p53 and RFX7 are required to balance mTORC1 activity. Uninduced p53 and RFX7 are required to limit mTORC1 activity under physiological nutrient abundance, but are insufficient to keep high or hyper-activated mTORC1 in check. The activation of p53-RFX7 signaling enables p53 and RFX7 to reduce the activity of hyper-active mTORC1.

Standard cell culture media, including the Dulbecco’s Modified Eagle Medium (DMEM), contain a non-physiological excess of nutrients, including glucose and glutamine. At the same time they are low on uric acid [34]. Previous studies on p53-mediated AKT and mTOR control employed standard cell culture media containing an excess in most nutrients [4–6, 8, 9]. Nutrient availability, however, is a most critical factor for the regulation of mTORC1 [1] calling for a careful consideration of culture conditions. In line with mTORC1’s intricate connection to nutrient-sensing pathways, Thr389 phosphorylation of S6K was lower when U2OS cells were cultured in Human Plasma Like Medium (HPLM) [34] instead of DMEM (Fig. 3A). When cultured in HPLM and irrespective of any treatment, the glucose-sensing AMPK was strongly activated. Most importantly, RFX7 and p53 were required to sustain low mTORC1 activity irrespective of Nutlin-3a treatment when U2OS were cultured in HPLM (Fig. 3A). Notably, increased mTORC1 activity upon p53 or RFX7 depletion was not associated with decreased AMPK activity (Fig. 3A), suggesting that p53 and RFX7 control mTORC1 largely through alternative pathways. While RFX7 was required to sustain low mTORC1 activity also in HPLM-cultured RPE-1 and HCT116 cells, a universal contribution by p53 could not be observed (Fig. 3B). Interestingly, Nutlin-3a treatment did not lead to a further reduction in pThr389-S6K levels when cells were cultured under physiological nutrient abundance, suggesting that p53 and RFX7 can reduce mTORC1 activity only to a certain extent (Fig. 3A and B). Next, we sought to identify the nutrients that were most critical to the observed changes. While the formulation of HPLM differs largely from DMEM [34], mTORC1 has long been known to be particularly sensitive to glucose and glutamine availability. HPLM contains 5 mM glucose and 0.55 mM glutamine compared to 25 mM glucose and 3.97 mM glutamine in DMEM [34]. Previously, a simple ‘physiological DMEM’ formulation has been described that contains 5 mM glucose, 0.5 mM glutamine, and 5% FBS [35]. Intriguingly, results obtained with this DMEM^physio^ largely mirrored the results we obtained with HPLM, indicating that glucose and glutamine levels were particularly important for p53-RFX7-dependent mTORC1 inhibition (Fig. 3C). Similar to U2OS cultured in HPLM, U2OS cultured in DMEM^physio^ displayed substantially reduced mTORC1 activity and activated AMPK. Importantly, p53 and RFX7 were required to sustain physiologically low mTORC1 activity also in DMEM^physio^ cultured cells, indicating that uninduced p53 and RFX7 activity played an important role (Fig. 3C). Together, our investigation reveals a previously unknown role of p53 and RFX7 in limiting mTORC1 activity under physiological nutrient abundance.

## Discussion

It is long known that p53 can inhibit mTORC2-AKT and mTORC1 signaling [4, 5, 8], but deciphering the underlying mechanisms proved to be challenging. While many components and regulators of the PI3K-AKT-mTORC1 and AMPK-mTORC1 signaling pathways are regulated by p53 on mRNA level, only a few proteins were shown so far to play a role in p53-mediated AKT and mTORC1 inhibition. For example, *SESN1* and *SESN2* are direct p53 targets and their encoded proteins emerged as crucial effectors in p53-mediated mTORC1 inhibition [6]. The vast network that regulates mTORC1 activity encompasses cross-talk and feedback mechanisms, and it is the complexity of mTORC1 regulation that impedes our understanding of how the central tumor suppressor p53 takes control of this crucial survival kinase.

Here, we identify RFX7 as the first protein required for p53 to inhibit both AKT and mTORC1. We establish p53-RFX7-DDIT4 as a signaling axis inhibiting mTORC2-dependent AKT activation (Fig. 2D), but the mechanism through which DDIT4 affects Ser473 phosphorylation of AKT remains to be uncovered. Our findings show RFX7 to be a regulator of AKT and mTORC1 activity both downstream and independent of p53. Intriguingly, AKT activity measured by Ser473 phosphorylation was not strictly linked to mTORC1 activity (Fig. 2A and B), suggesting that threshold-driven switches may play an important role in AKT-mTORC1 signaling. In agreement with an earlier study [11], our data indicate that activating AMPK is not critical for p53 or RFX7 to inhibit mTORC1. Yet, p53 may affect AMPK function through increased pSer182-AMPKβ levels (Fig. 2C), which are associated with nuclear export of AMPK [36]. Investigating further RFX7 targets may reveal additional factors involved in p53 and RFX7-dependent regulation of mTORC1 signaling. Notably, RFX7 abundance was reduced upon treatment with Actinomycin D (Fig. 1D), depletion of DDIT4 (Fig. 2A and B), and under physiological nutrient abundance (Fig. 3A and C) indicating that feedback mechanisms may balance RFX7 levels to avoid shortage and oversupply. Based on our results comparing physiological and excess nutrient abundance, we propose a phase model of p53-RFX7-mediated mTORC1 inhibition that depends on nutrient-associated mTORC1 activity (Fig. 3D). These findings may serve as a starting point to uncover further context-dependent mechanisms of p53 in controlling AKT and mTOR.

## Methods

### Cell culture, drug treatment, and transfection

U2OS and HCT116 cells (ATCC, Manassas, Virginia, USA) were grown in high glucose Dulbecco’s modified Eagle’s media (DMEM) with pyruvate (Thermo Fisher Scientific, Darmstadt, Germany). RPE-1 hTERT cells (ATCC) were cultured in DMEM:F12 media (Thermo Fisher Scientific). Culture media were supplemented with 10% fetal bovine serum (FBS; Thermo Fisher Scientific) and penicillin/streptomycin (Thermo Fisher Scientific). Alternatively, U2OS cells were cultured in human plasma like medium [34] (HPLM; Thermo Fisher Scientific) supplemented with 10% dialyzed, heat-inactivated FBS (Thermo Fisher Scientific) or in DMEM^physio^ made of no glucose, no glutamine DMEM (Thermo Fisher Scientific) supplemented with 5% FBS, 5 mM glucose, and 0.5 mM glutamine (Thermo Fisher Scientific) [35]. Cell lines were tested twice a year for *Mycoplasma* contamination using the LookOut Detection Kit (Sigma), and all tests were negative.

Cells were treated with DMSO (0.15%; Carl Roth, Karlsruhe, Germany), Nutlin-3a (10 µM; Sigma Aldrich, Darmstadt, Germany), Actinomycin D (5 nM; Cayman Chemicals, Ann Arbor, Michigan, USA), or Doxorubicin (1 µM; Cayman Chemicals) for 24 h. For knockdown experiments, cells were seeded in six-well plates or 6 cm dishes and reverse transfected with 5 nM Silencer Select siRNAs (Thermo Fisher Scientific) using RNAiMAX (Thermo Fisher Scientific) and Opti-MEM (Thermo Fisher Scientific) following the manufacturer protocol. The following siRNAs were used (Thermo Fisher Scientific): siControl (#4390844). siControl#2 (#4390846), siTP53 (#s607), siRFX7 (#s35057), siRFX7#2 (#s35059), siDDIT4 (#s29166).

### Chromatin immunoprecipitation, RNA extraction, and reverse transcription semi-quantitative real-time PCR (RT-qPCR)

ChIP was performed with the SimpleChIP Kit (Cell Signaling Technology, Canvers, MA, USA) following the manufacturer instructions. 3 µg of p53 (kind gift from Dr. Bernhard Schlott [37]) or RFX7 (#A303-062A Bethyl Laboratories, Montgomery, TX, USA) antibody were used per IP. Sonication was performed on a Bioruptor Plus (Diagenode, Seraing, Belgium). ChIP-qPCR was performed with a Quantstudio 5 (Thermo Fisher Scientific) using Power SYBR Green MasterMix (Thermo Fisher Scientific) following the manufacturer protocol. The following ChIP-qPCR primers were used: *GAPDH* (#4471, Cell Signaling Technology), *MDM2* (forward: TCGGGTCACTAGTGTGAACG, reverse: TGAACACAGCTGGGAAAATG), and *DDIT4* (forward: GTTCGACTGCGAGCTTTCTG, reverse: GCCTTGGCCAATGGACTC).

Total cellular RNA was extracted using the RNeasy Plus Mini Kit (Qiagen, Hilden, Germany) following the manufacturer protocol. One-step reverse transcription and real-time PCR was performed with a Quantstudio 5 using Power SYBR Green RNA-to-CT 1-Step Kit (Thermo Fisher Scientific) following the manufacturer protocol. We identified *ACTR10* as a suitable control gene that is not regulated by p53 but expressed across 20 gene expression profiling datasets [32]. The following RT-qPCR primers were used: *ACTR10* (forward: TCAGTTCCGGAAGGTGTCTT, reverse: GGACGCTCATTATTCCCATC), *DDIT4* (forward: AGACACGGCTTACCTGGATG, reverse: CATCAGGTTGGCACACAAGT).

### Western blot analysis

Cells were lysed in IP lysis buffer (Thermo Fisher Scientific) containing protease and phosphatase inhibitor cocktail (Roche, Grenzach-Wyhlen, Germany or Thermo Fisher Scientific). Protein lysates were scraped against Eppendorf rack for 20 times and centrifuged with 15000 *rpm* for 15 min at 4 °C. The protein concentration of supernatant lysates was determined using the Pierce 660 nm Protein Assay Kit (Thermo Fisher Scientific) and a NanoDrop ND1000 Spectrophotometer (Thermo Fisher Scientific). Proteins were separated in a Mini-Protean TGX Stain-Free Precast 4–15% Gel (Bio-Rad) using Tris/Glycine/SDS running buffer (Bio-Rad). Proteins were transferred to a 0.2 µm or a low-fluorescence 0.45 µm polyvinylidene difluoride transfer membrane either using a Trans-Blot Turbo Mini Transfer Pack (Bio-Rad) in a Trans-Blot Turbo (Bio-Rad) or using a Mini Trans-Blot Cell (Bio-Rad) in a Mini-Protean Tetra Cell (Bio-Rad). Following antibody incubation, membranes were developed using Clarity Max ECL (Bio-Rad) and a ChemiDoc MP imaging system (Bio-Rad) or, alternatively, ChemiDoc MP’s fluorescence detection was used.

Antibodies and their working concentrations: anti-mouse (1:5000; #31430, Thermo Fisher Scientific, or 1:5000, #7076, Cell Signaling Technology, or 1:10000, #12005867, Bio-Rad), anti-rabbit (1:5000; #7074, Cell Signaling Technology or 1:10000, #12004162, Bio-Rad), actin (1:5000; #MA1-140, Thermo Fisher Scientific), RFX7 (1:1000; #A303-062A, Bethyl Laboratories), p53 (1:2000; kind gift from Bernhard Schlott [37]), DDIT4 (1:1000; #10638-1-AP, Proteintech), pThr389-p70S6K (1:1000; #9234, Cell Signaling Technology), p70S6K (1:1000; #9202, Cell Signaling Technology), pSer473-AKT (1:1000; #4060, Cell Signaling Technology), AKT (1:1000; #9272, Cell Signaling Technology), pThr172-AMPKα (1:1000; #2535, Cell Signaling Technology), AMPKα (1:1000; #5831, Cell Signaling Technology), pSer182-AMPKβ1 (1:1000; #4186, Cell Signaling Technology), AMPKβ1/2 (1:1000; #4150, Cell Signaling Technology), and pSer79-ACC (1:1000; #11818, Cell Signaling Technology).

### Statistics

ChIP-qPCR data were analyzed using a two-sided unpaired *t*-test. Bar graphs display mean and standard deviation. *, **, ***, and n.s. indicate *p* values < 0.05, <0.01, <0.001, and >0.05, respectively. The number of replicates is indicated in each Figure legend. The experiments were not randomized and investigators were not blinded to allocation during experiments.

## Supplementary information

Supplementary Figure 1

## Data Availability

RFX7 ChIP-seq data is accessible through GEO series accession number GSE162184 [31]. p53 ChIP-seq data was obtained from CistromeDB [38], IDs 82544 [39], and 33077 [40]. Source data for Figures are available from the corresponding authors upon request.
