# Supplementary material for: p53-mediated AKT and mTOR inhibition requires RFX7 and DDIT4 and depends on nutrient abundance
Source: Oncogene. 2021 Dec 14;41(7):1063–9. doi: 10.1038/s41388-021-02147-z (PMC8837532; doi:10.1038/s41388-021-02147-z)
Supplement: Supplementary file 1 — Supplementary Figure 1 [file 41388_2021_2147_MOESM1_ESM.pdf]

Supplementary Figure 1

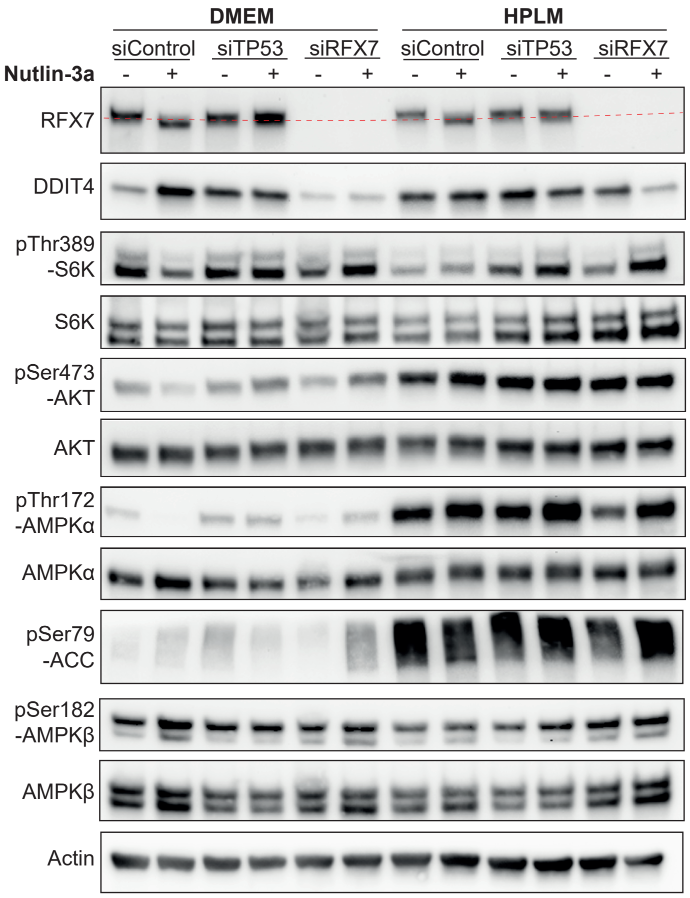

Supplementary Figure 1. Western blot analysis of U2OS cells transfected with indicated siRNAs, treated with 10 μM Nutlin-3a or DMSO control, and cultured in DMEM and HPLM.
